# Environmental Epigenomes

**DOI:** 10.3390/epigenomes7030021

**Published:** 2023-09-07

**Authors:** Bambarendage P. U. Perera, Frédéric Silvestre

**Affiliations:** 1 Department of Environmental Health Sciences, School of Public Health, University of Michigan, 1415 Washington Heights, Ann Arbor, MI 48109, USA; 2 Laboratory of Evolutionary and Adaptive Physiology, Institute of Life, Earth and Environment, University of Namur, 5000 Namur, Belgium; frederic.silvestre@unamur.be

**Keywords:** environmental epigenetics, toxicology, DNA methylation, population epigenetics, toxicoepigenetics, ecoepigenetics, biomarkers, breast cancer, cardiometabolic health

## Abstract

Research in epigenetics has dramatically risen during the last decade to include aspects of environmental biology. However, many questions remain regarding the effects of environmental stressors on the epigenome, incorporating the particular role of epigenetic mechanisms in the adaptation and evolution of organisms in changing environments. Epigenetics is commonly defined as mitotically and/or meiotically heritable changes in gene function that occur without altering the underlying DNA sequence. It encompasses DNA (hydroxy)methylation, histone modifications, chromatin structure, and non-coding RNAs that may be inherited across generations under certain circumstances. Epigenetic mechanisms are perfect candidates to extend our understanding of the impact of environmental stressors on organisms and to explain the rapid phenomenon of adaptive evolution. Existing evidence shows that environmental cues can affect the epigenome and modify gene expression accordingly. These changes can then induce phenotypic modifications that are morphological, physiological, or behavioral at the organismal level. In this Special Issue focusing on environmental epigenetics, we provide an overview of influences to the epigenome that are driven by various environmental and evolutionary factors, with a particular focus on DNA methylation (DNAm). Five research groups have contributed insightful studies or reviews on (1) DNAm and demethylation events affected by the exposome; (2) DNAm as a potential biomarker to determine cardiometabolic risk early in life; (3) consequences of DNAm across multiple generations; (4) DNAm variation within natural animal populations; and (5) epigenetic mechanisms in genetically uniform organisms. Collectively, the articles from this Special Issue consistently support that environmental changes can induce long-lasting epigenetic effects within a given organism pertaining to individual risk for disease, or multi-generational impacts that ultimately impact evolution.

## 1. Introduction

Nature versus nurture argues that the fitness of an individual is primarily driven by either their genetic makeup or their environment. The concept of epigenetics greys the boundary on this debate by clarifying how environmental factors such as climate, nutrition, stress, and toxicants influence gene expression, and thus biological processes, without altering the underlying DNA sequence [1]. DNA methylation (DNAm) at cytosine residues, histone tail modifications, chromatin architecture, and non-coding RNA constitute reversible epigenetic modifications involved in modulating gene expression [2]. The environmental perturbations on the epigenome can be detrimental to health and survival. However, these consequences may be reversed through environmental or biological interventions with a priori knowledge of the epigenetic mechanisms related to molecular and evolutionary adaptations in the changing environment [3,4].

Phenotypic variation is a key element in populations to permit adaptation and evolution to environmental changes. It has been acknowledged that phenotypic variation can arise from either genetic variation or phenotypic plasticity, wherein the same genome exhibits different expressions in response to environmental cues. However, emerging evidence suggests that epigenetic mechanisms can also underlie phenotypic plasticity [5]. Epigenetics is commonly defined as the heritable changes in gene expression that occur mitotically and/or meiotically and are not attributable to gene sequence alterations [6]. Importantly, these epigenetic changes may be inherited across generations under certain circumstances to contribute to the transmission of phenotypic traits [7]. It is now widely recognized that epigenetic modifications, akin to genetic changes, play a critical role in short-term microevolutionary processes while also contributing to macroevolutionary phenomena.

This Special Issue on environmental epigenetics includes a total of five articles, with two original studies and three insightful reviews. Collectively, the two original studies indicate how environmental exposure to chemicals influence DNAm-directed mechanisms and human health.

In addition to epigenetic dysregulation, DNA damage and mutagenesis are extensively studied for their oncogenic effects. The deamination of 5-methyl-cytosine (5mC) to thymine (C > T) can occur actively or passively via DNA (de)methylation or hydrolytic deamination, respectively. It is also a hallmark mutational signature in human cancers, including breast cancer. Partner and localizer of BRCA2 (*PALB2*) is a gene known to be disrupted in breast cancer and also contains C > T mutations. Courant and colleagues highlighted a proof of concept supporting that environmental exposures can guide DNA (de)methylation reactions to instigate C > T mutations. They utilized a mammary epithelial cell model (MCF10A) coupled with environmental exposures, including folate, Diuron, glyphosate, perfluorooctanoic acid (PFOA), iron, zinc, and ascorbic acid, to determine that exposomes can generate C > T mutations effecting DNA (de)methylation at *PALB2*: (https://www.mdpi.com/2075-4655/6/4/32, accessed on 11 February 2023).

Epigenetic modifications such as DNAm may serve as biomarkers, which are often used for the early detection of diseases or as determinants of disease risk. These modifications can further be interrogated to assess disease-associated mechanisms for potential interventions. In previous reports, DNAm changes have been associated with cardiovascular disease, specifically during adulthood. However, it remains unclear how environmental perturbations can impact cardiometabolic diseases during the susceptible life stage of adolescence. To answer this question, Aljahdali and colleagues utilized early childhood and adolescent blood leukocyte DNAm as a determinant of cardiometabolic health from the Early Life Exposure in Mexico to Environmental Toxicants (ELEMENT) birth cohort. They quantified DNAm changes at the long interspersed nuclear element-1 (*LINE-1*) repeat element, as well as the maternally imprinted transcript *H19*, 11-hydroxysteroid dehydrogenase type 2 (*11b-HSD-2*) and peroxisome proliferator-activated receptor alpha (*PPAR-*⍺). The authors concluded that DNAm may serve as an indicator for early life risk for cardiometabolic abnormalities in specific genomic loci related to *LINE-1* and *11b-HSD-2*: (https://www.mdpi.com/2075-4655/7/1/4, accessed on 11 February 2023).

In recent years, there has been a notable surge in research interest in the intraspecific spatial distribution of epigenetic marks, with a particular focus on DNAm. Within this Special Issue, Chapelle and Silvestre conduct a comprehensive review of DNAm variation observed in natural animal populations. They consider the hypothesis that epigenetic variation is independent from genetic variation and explore how DNAm dynamics contribute to the establishment of population structure. They present compelling arguments on the significant roles played by epigenetics in reshaping traditional evolutionary theories. By examining the relationship between genetics and epigenetics, their review enhances our understanding of the factors that influence evolutionary processes within natural animal populations. As a result, it paves the way for further investigations and advancements in the field of epigenetics, ultimately enriching our comprehension of the complexities of evolutionary biology: (https://www.mdpi.com/2075-4655/6/4/31, accessed on 11 February 2023).

To comprehensively assess the potential impact of epigenetics on adaptation and evolution, it is crucial to understand its relationship with genetic variation. To date, only a limited amount of empirical research has been dedicated to disentangling epigenetic from genetic variation. One promising approach to this challenge is the study of genetically uniform populations, which are more commonly found in asexually reproducing organisms. In this context, Vogt’s review within the current Special Issue emerges as a pivotal contribution and highlights the fundamental role played by epigenetic variation in the adaptive capacity of genetically uniform organisms across diverse environments. Vogt’s review draws from a wide array of examples spanning bacteria, protists, fungi, plants, and animal populations to illustrate how epigenetic mechanisms facilitate environmental adaptation in these organisms. Moreover, the author delves into the mechanistic explanations of both the general purpose genotype hypothesis and the genetic paradox of invasions, suggesting that epigenetic processes can contribute to these phenomena: (https://www.mdpi.com/2075-4655/7/1/1, accessed on 11 February 2023).

The stability of epigenetic marks across generations is a crucial aspect to comprehend the roles of epigenetics in the adaptation and evolution of populations. In this current Special Issue, Hanson and Liebl’s review comprehensively evaluates the implications of DNAm both within individual generations and in its transgenerational effects. One intriguing and relatively underexplored area of DNAm is its mutagenic potential and capacity to induce rapid evolutionary changes. The authors explore how DNAm can influence DNA mutation rates in a more directed and accelerated manner compared to classical stochastic genetic mutations alone. By shedding light on the mutagenic properties of DNAm, Hanson and Liebl’s review opens new avenues for understanding how epigenetic mechanisms can contribute to the rapid and directed evolution of populations: (https://www.mdpi.com/2075-4655/6/4/33, accessed on 11 February 2023).

Taken together, all five articles in this Special Issue emphasize the critical need to interrogate epigenetic mechanisms of intrinsic and extrinsic stimuli that predict the health of individuals later in life, as well as populations throughout evolution.
